# H3K27M-mutant, hemispheric diffuse glioma in an adult patient with prolonged survival

**DOI:** 10.1093/noajnl/vdab135

**Published:** 2021-09-17

**Authors:** Noel Chia, Andrea Wong, Kejia Teo, Ai Peng Tan, Balamurugan A Vellayappan, Tseng Tsai Yeo, Shoo Yi Oh, Char Loo Tan

**Affiliations:** 1 Department of Pathology, National University Health System, Singapore; 2 Department of Medical Oncology, National University Health System, Singapore; 3 Division of Neurosurgery, Department of Surgery, National University Health System, Singapore; 4 Department of Diagnostic Imaging, National University Health System, Singapore; 5 Department of Radiation Oncology, National University Health System, Singapore

**Keywords:** H3K27M, diffuse glioma, hemispheric, *ATRX*, adult

The K27M mutation of the histone 3 (H3) isoforms has been identified as the main driver mutation of diffuse gliomas arising from the midline structures. This tumor predominantly affects children^1^ and to a lesser extent the adult population.^2–4^ Pediatric patients with H3K27M-mutant diffuse midline glioma (DMG) have a uniformly poor prognosis, with a median overall survival of 9 to 15 months,^1^ while the prognosis of adult patients is more variable.^2–5^ Exceedingly rare, H3K27M mutation has been identified in diffuse hemispheric gliomas with only a handful of reported cases to date.^6–8^ We present a unique case of an adult patient with hemispheric, histologically high-grade, diffuse glioma harboring H3K27M mutation, with prolonged survival. In addition, we performed a literature review of such cases.

## Clinical Presentation

**Figure 1.**
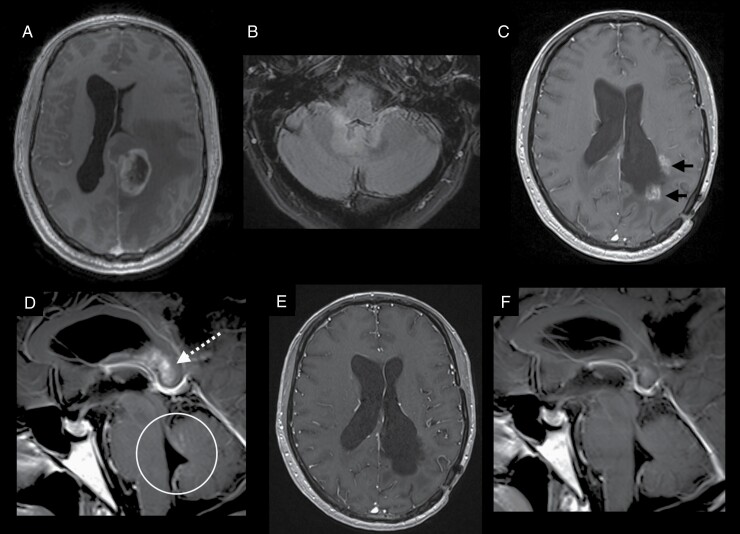
MRI brain images. (A) Axial T1 post-contrast image at the time of diagnosis showed an irregular rim enhancing lesion in the left fronto-parietal lobe with marked surrounding vasogenic edema. (B) Axial T2 FLAIR post-contrast image 22 months later showed new FLAIR hyperintense lesion around the fourth ventricle, suspicious for tumor recurrence. (C) Axial and (D) sagittal T1 post-contrast images (37 months from the time of diagnosis) showed enhancing lesions around the surgical cavity (black arrows), extending into the splenium of the corpus callosum (white dotted arrow). Leptomeningeal and ependymal enhancements were also observed within the posterior fossa, centered around the fourth ventricle (white circle). (E,F) Follow-up MRI brain 3 months after the initiation of intravenous bevacizumab showed near complete resolution of the previously seen enhancing lesions.

**Figure 2.**
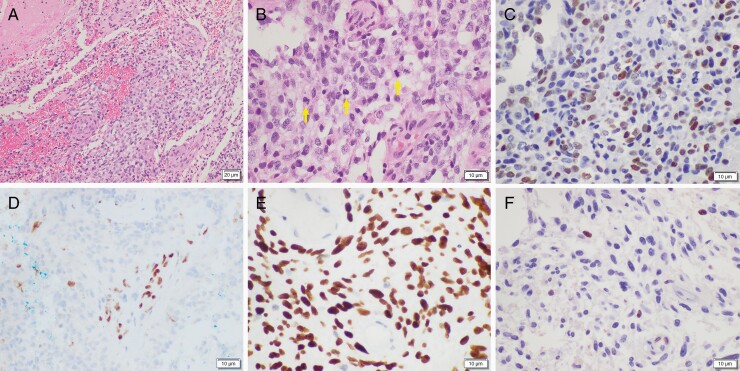
Histologic and immunohistochemical findings of the tumor. (A) Hematoxylin- and eosin-stained image showed a highly cellular tumor with necrosis (scale bar = 20 µm). (B) The tumor showed marked nuclear atypia. Numerous mitotic figures were also present (arrows). Tumor cells showed positive expression for (C) OLIG2, (D) loss of nuclear expression for ATRX with positive internal control, (E) strong positive nuclear expression for H3K27M with negative internal control, and (F) reciprocal loss of H3K27me3 with positive internal control (B–F: scale bar = 10 µm).

The patient is a 41-year-old male with no known medical illness, presented with a 3-month history of unabating generalized headache and progressive right-sided weakness. Physical examination showed weakness (power 3 on the Medical Research Council’s scale) of the right upper and lower limbs. Magnetic resonance imaging (MRI) of the brain revealed a 3.4 cm irregular rim-enhancing mass in the left fronto-parietal lobe with extensive peri-lesional edema (Figure 1A). There was no extension into the ventricles, involvement of the thalamus or corpus callosum at the time of initial diagnosis. He subsequently underwent gross total resection of the tumor under awake mapping. Histology showed an infiltrative, cellular glial tumor with nuclear atypia, microvascular proliferation, and necrosis (Figure 2A and B). Mitotic count of up to 15 per 10 high power fields were seen (Figure 2B). The tumor cells were positive for GFAP and OLIG2 (Figure 2C). They were negative for IDH1 R132H mutant protein, demonstrated loss of ATRX nuclear expression (Figure 2D) and exhibited 5–10% expression for p53. In view of the young age of the patient, negative IDH1 R132H mutant protein and loss of ATRX expression in the tumor cells, *IDH1/2* Sanger sequencing was performed to look for a noncanonical IDH mutation. The result, however, returned as wildtype for IDH mutation. H3K27M and H3K27me3 immunostains were performed for completeness. Interestingly, the tumor cells revealed diffuse and strong nuclear reactivity for H3K27M (Figure 2E), with reciprocal loss of nuclear expression for H3K27me3 (Figure 2F). *MGMT* promoter was not methylated. FISH for *CDKN2A/B* was negative for homozygous deletion. A diagnosis of high-grade glioma, IDH-wildtype, H3K27M-mutant was made with a comment that the significance of H3K27M mutation in a hemispheric glioma is uncertain in view of paucity of data. Following the diagnosis, the patient underwent adjuvant treatment according to the Stupp protocol—completing radiotherapy (RT) 60Gy in 30 fractions with concurrent temozolomide (TMZ), followed by another 12 cycles of adjuvant TMZ.

Twenty-two months from the initial diagnosis, MRI brain showed post-contrast fluid-attenuated inversion recovery (FLAIR) hyperintensity around the fourth ventricle (Figure 1B). This was radiologically in keeping with tumor recurrence. As the recurrence occurred outside of the region where high-dose RT was previously given, the patient underwent salvage treatment with RT (57 Gy in 30 fractions) and concurrent TMZ, followed by TMZ alone. Unfortunately, the disease progressed shortly after completion of treatment (37 months from the initial diagnosis). MRI showed enhancing foci around the previous surgical cavity (Figure 1C). Enhancing lesions were also seen extending into the splenium of the corpus callosum. Of note, there was leptomeningeal enhancement within the posterior fossa (Figure 1D), but not involving the spine. At this juncture, he was noted to have mild cognitive decline and increasing unsteadiness of gait. He was started on oral lomustine 90 mg/m^2^ 6 weekly and intravenous bevacizumab 15 mg/kg 3 weekly. Follow-up MRI brain performed 3 months later showed near complete resolution of the previously seen enhancing lesions, likely related to the anti-angiogenic treatment (Figure 1E and 1F). The patient is now 42 months from the initial diagnosis, and remains well on this current line of treatment. Comprehensive molecular profiling of the tumor, using next-generation sequencing method, 3 years after the initial diagnosis was unfortunately unsuccessful.

## Discussion

The molecular landscape of adult-type diffuse gliomas is characterized by mutation involving the *TERT* promoter leading to telomerase activation or an alternative lengthening of telomere phenotype (ALT), most commonly attributed to *ATRX* mutation, and less commonly due to *H3* mutation.^9,10^ While H3G34R/V is a common mutation implicated in hemispheric gliomas in the spectrum of H3-mutant gliomas,^11^ the positivity for OLIG2 and lack of p53 overexpression seen in our case make it less likely to be H3G34-mutant. It was surprising to note the unequivocal positive H3K27M staining in the tumor cells. Similar to findings from large scale molecular analyses of H3K27M-mutant DMGs, the tumor in our case did not show *MGMT* promoter methylation or homozygous *CDKN2A/B* deletions.^1^

The diagnostic criteria of DMG, H3K27M-mutant was refined by the Consortium to Inform Molecular and Practical Approaches to CNS Tumor Taxonomy—Not Official WHO (cIMPACT-NOW) in update 2,^12^ mandating the need for such tumors to have diffuse histologic growth pattern, involve midline structures, and demonstrate mutation for H3K27M via immunohistochemistry (IHC) or molecular sequencing. The involved midline locations are more commonly the brainstem, thalamus, spinal cord, or less commonly the third ventricle, hypothalamus, cerebellum and pineal region. There are only 8 cases of H3K27M-mutant diffuse gliomas involving the non-midline structures in the literature, including the case we presented herein, to the best of our knowledge (Table 1).^6–8^ Such cases tend to involve the adult population, with age ranging from 20 to 76 years old and a mean age of 47 years old. There is no gender predilection (4 females and 4 males). The tumor locations include frontal and temporal lobe (*n* = 3), insular cortex (*n* = 1), and the corpus callosum (*n* = 4). No tumor multifocality or involvement of any of the aforementioned midline structures were reported in all of the cases. Four cases were histologically grade 4, 3 cases were histologically grade 3, and 1 case was histologically grade 2. All cases were proven IDH-wildtype and H3K27M-mutant by IHC or sequencing. Loss of ATRX staining in the tumor cells was seen in 2 cases and intact ATRX staining in the remaining cases. Of note, in the case by López et al., despite an intact ATRX staining, *ATRX* mutation was identified in sequencing study.^8^ P53 immunostain was overexpressed in 4 cases. Survival data was only available in 7 patients (range 3–42 months, median 10.8 months). Three patients were last seen alive with follow-up ranging from 6.6 to 42 months, including our patient who has demonstrated the longest survival thus far. Four other patients passed away with a median overall survival of 7.8 months (range 3–21.9 months). Interestingly, all the patients who succumbed to the disease had tumor involving the corpus callosum. While the corpus callosum is not a predefined midline structure in the diagnostic criteria for “DMG, H3K27M-mutant” in the revised 4th edition of WHO Classification of the Central Nervous System Tumors or cIMPACT-NOW update 2, our analysis suggests that corpus callosum involvement appears to be a negative prognostic factor in the spectrum of H3K27M-mutant diffuse gliomas. This may be related to the limited resectability of tumors involving the corpus callosum.

**Table 1.** Summary of Cases of Hemispheric Diffuse Gliomas With H3K27M Mutation from the Various Publications^6–8^

No.	Paper	Age (yr)	Sex	Tumor Location	Histology and Grade	Immunohistochemistry	Molecular Data	Extent of Surgery	Adjuvant Treatment	Disease Course	Follow-up (months)	Outcome
1	López et al.	20	F	Left insular cortex	Diffuse astrocytic neoplasm, Grade II	IDH1R132H negative H3K27M positive ATRX retained	IDH wildtype H3K27M mutated ATRX mutated EPB41L2-BRAF fusion TP53 wildtype EGFR wildtype	Gross total resection	No data	No data	No data	No data
2	La Rocca et al.	48	F	Corpus callosum (splenium)	Diffuse astrocytic neoplasm, Grade IV	IDH1R132H negative H3K27M positive ATRX retained P53 (5-10%)	Nil	Biopsy	Concurrent radiotherapy and systemic chemotherapy including TMZ	Passed away before completing treatment	3	Died
3	Wang et al.	44	M	Temporal lobe	AA, Grade III	IDH1R132H negative H3K27M positive ATRX retained P53 mutated	IDH wildtype	Resection	No data	No data	32.8	Alive
4	Wang et al.	44	M	Frontal lobe	AA, Grade III	IDH1R132H negative H3K27M positive ATRX retained P53 mutated	IDH wildtype	Biopsy	No data	No data	6.6	Alive
5	Wang et al.	45	F	Corpus callosum	GBM, Grade IV	IDH1R132H negative H3K27M positive ATRX loss P53 negative	IDH wildtype	Resection	No data	No data	10.8	Died
6	Wang et al.	60	F	Corpus callosum	GBM, Grade IV	IDH1R132H negative H3K27M positive ATRX retained P53 mutated	IDH wildtype	Resection	No data	No data	4.8	Died
7	Wang et al.	76	M	Corpus callosum	AO, Grade III	IDH1R132H negative H3K27M positive ATRX retained P53 mutated	IDH wildtype	Biopsy	No data	No data	21.9	Died
8	Chia et al.	41	M	Left fronto-parietal lobe	High-grade glioma, IDH-wildtype, H3K27M-mutant	IDH1R132H negative H3K27M positive ATRX loss P53 (5-10%)	IDH wildtype MGMT-p unmethylated No homozygous deletions for CDKN2A/B (FISH)	Gross total resection	Completed concurrent TMZ/radiation, adjuvant TMZ, lomustine and bevacizumab	Relapse at 22 and 37 months from initial diagnosis	42	Alive

AA, anaplastic astrocytoma; AO, anaplastic oligodendroglioma; F, female; M, male; TMZ, temozolomide; GBM, glioblastoma; yr, year.

Despite 2 episodes of tumor relapse, it is intriguing to observe that our patient has demonstrated prolonged survival, much longer than the pediatric or adult patients with H3K27M-mutant DMGs and adult patients with IDH-wildtype glioblastomas (median overall survival in published series: 11.2 months, 9.3–27.6 months, and 13.9–17 months, respectively).^1,3–5,13^ While DMGs may have limited surgical options due to involvement of critical structures such as the brainstem and spinal cord, a tumor present at a hemispheric location may be more amenable to surgical resection, which may have contributed to the better clinical outcome observed in our case. Interestingly, a recent study on H3K27M-mutant DMGs in adults have found divergent clinical and genetic profiles compared to pediatric tumors, such as a higher predilection of thalamic and spinal cord involvement, K27M mutation exclusively in the *H3-3A* gene, lower incidence of *PDGFRA* amplification, and absence of *ACVR1* mutation in the adult patients.^3^ In this study, there was better overall survival in adults compared to both pediatric H3K27M-mutant DMGs and adult IDH-wildtype glioblastoma, questioning the aggressive behavioral role ascribed to H3K27M mutation in adult patients. On the other hand, the clinical and molecular characteristics of adult H3K27M-mutant DMGs also differ from IDH-wildtype glioblastomas, the former having an earlier age of diagnosis, exclusive midline location, and less frequent methylation of *MGMT* promoter and no *EGFR* amplification.^3,4^ Recent studies have also identified *CDKN2A/B* homozygous deletions as a frequent event and an independent poor prognostic factor in IDH-wildtype glioblastoma.^14^ The lack of homozygous *CDKN2A/B* deletions could have, in part, contributed to the prolonged survival of our patient. Overall, while the role of H3K27M mutation in adult gliomas remains to be seen, the current data suggests it may represent a distinct neoplasm, as opposed to the pediatric type, H3K27M-mutant DMGs or the adult-type IDH-wildtype glioblastomas.

In summary, we present a rare case of a hemispheric located, histologically high grade, H3K27M-mutant diffuse glioma in an adult patient with prolonged survival. As the biology and clinical outcome of such an unusual tumor remains poorly understood to date, it may be best to classify such these tumors as “not elsewhere classified” according to cIMPACT-NOW update 1.^15^ In the unusual setting of loss of ATRX staining in tumor cells in an IDH-wildtype, hemispheric tumor, we recommend H3K27M testing to identify more of such cases for further prognostic stratification.
